# A System of Medicine

**Published:** 1899-03

**Authors:** 


**A System of Medicine.** By many Writers. Edited by THOMAS CLIFFORD ALLBUTT, M.D., F.R.S. Vol. V. Pp. xii., 1058. London: Macmillan and Co. Limited. 1898.

An exhaustive account of the diseases of the respiratory organs and of the circulatory system fills up this volume. It is somewhat belated, having waited for Professor Welch's contribution on thrombosis and embolism, which after all did not arrive in time; nevertheless, it well maintains the standard of the four previous volumes.

Dr. William Ewart writes the articles on bronchitis and bronchiectasis, in which many new methods of treatment are described; the most noteworthy of these is the creasote method of Dr. Arnold Chaplin, whose object is to bring about a complete expectoration of the bronchial contents, the vapour inhaled being concentrated to an almost intolerable degree. Whilst the cavities are being cleared and disinfected collateral expansion of the lung is induced by the cough, and the gradual contraction of the sacculations is promoted. This inhalation method has given such brilliant results, that every sufferer should have the benefit of a trial. Treatment by intra-tracheal injection of menthol and guaiacol in olive oil has given good results in inveterate cases of fœtid bronchiectasis, but Dr. Ewart gives a caution as to its use in phthisis. Amongst other methods of treatment are mentioned "(*a*) The inhalation of an oxygenated and terebinthinated atmosphere; (*b*) systematic exercise, at first passive only, of the thoracic muscles and of the abdominal muscles, including the use of dumb-bells or clubs, and a variety of postural exercises; (*c*) systematic respiratory gymnastics, such as deep inspirations followed by deep expirations in various attitudes, reading aloud or singing; (*d*) general massage and passive resistance movements followed by brisk rubbing." It will be seen that Dr. Ewart's article is very complete in its scope, and gives much more encouragement to the chronic bronchial invalid than he commonly receives.

Dr. Pye-Smith's article on pneumonia, and Dr. Percy Kidd's on phthisis pulmonalis, give excellent summaries of what is established with regard to those diseases and their varieties. The former does not lead us to expect a great diminution in the present high mortality of pneumonia, for he remarks that "in the long run the expectant method of treatment, which interferes only as occasion requires, is followed by a far lower mortality than misplaced attempts to 'jugulate' the disease, or than a completely negative treatment." We can scarcely reconcile this statement with the results given by Petresco, whose name, spelt with a final u, does not yet appear to be as well known as his statistics deserve to be, inasmuch as he reports a mortality of 2 per cent. in contrast with the 25.5 per cent. from Guy's Hospital as given by the author in this volume.

A short chapter by Dr. H. D. Rolleston describes a comparatively little known variety of the pneumomycosis of Hughes Bennett, which bears the name "pulmonary aspergillosis," in which the destructive lesions in the lungs are due to their invasion by a fungus, the aspergillus fumigatus. In order to differentiate this malady from other forms of pseudo-tuberculosis it is necessary that cultures should be obtained; without these no opinion as to the identity of the form of aspergillus is valid. The clinical features may resemble either those of chronic pulmonary tuberculosis or those of emphysema, and a secondary infection with tubercle bacilli is not improbable. The prognosis is less grave than that of pulmonary tuberculosis, since the lesion is usually much slower, never sets up a general infection, and tends to undergo a gradual and spontaneous cure.

Dr. Kingston Fowler writes on emphysema and on syphilitic disease of the lungs, and Dr. James F. Goodhart on asthma and hay fever. The account of the diseases of the respiratory organs concludes with articles on intra-pleural tension, on pleurisy and pneumothorax, by Drs. Samuel West, Gee, Herringham, and Finlay.

Amongst the chapters on diseases of the circulatory system, that by the editor on the ubiquitous but little understood chlorosis is one of the most interesting; and other articles by the same author are on functional disorders and mechanical strain of the heart. He makes the remark that "the physician who cannot treat chlorosis successfully with iron should abandon the practice of medicine"; and he has often suspected that incurable chlorosis may mean insoluble pills. He believes that in the sound adult organism the effects of physical stress upon the heart are promptly counteracted by equilibrating machinery, and that the importance of muscular effort as a factor in cardiac disease has been much exaggerated.

